# Endothelial Notch Signaling Regulates the Function of the Retinal Pigment Epithelial Barrier via EC Angiocrine Signaling

**DOI:** 10.3390/antiox12111979

**Published:** 2023-11-07

**Authors:** Yali Niu, Yixuan Xi, Yutong Jing, Ziyi Zhou, Xiaojia Sun, Guoheng Zhang, Tianhao Yuan, Tianfang Chang, Guorui Dou

**Affiliations:** 1 Department of Ophthalmology, Eye Institute of Chinese PLA, Xijing Hospital, Fourth Military Medical University, Xi’an 710032, China; niuyali@stumail.nwu.edu.cn (Y.N.); 13572560975@163.com (Y.J.); zhouziyi@fmmu.edu.cn (Z.Z.); sunxiaojia@fmmu.edu.cn (X.S.); zhangguoheng2316@fmmu.edu.cn (G.Z.); ytianhao@fmmu.edu.cn (T.Y.); 2 College of Life Sciences, Northwest University, Xi’an 710069, China; xiyixuan@stumail.nwu.edu.cn

**Keywords:** RPE barrier function, EC angiocrine signaling, Notch signaling

## Abstract

The outer blood–retina barrier (oBRB), comprises tightly connected retinal pigment epithelium (RPE) cells, Bruch’s membrane, and choroid blood vessels, and is essential for retinal health and normal visual function. Disruption of the RPE barrier and its dysfunction can lead to retinal disorders such as age-related macular degeneration (AMD). In the present study, we investigated the essential role of choroid endothelial cells (ECs) in the RPE barrier formation process and its dysfunction. We discovered that ECs promoted RPE barrier formation through angiocrine signaling. Through blocking or activating endothelial Notch signaling and conducting experiments in vitro and in vivo, we confirmed that endothelial Notch signaling regulated the expression of heparin-binding epidermal growth factor (HBEGF) and consequently impacted the expression and activity of matrix metalloproteinases (MMP)-9 in RPE cells. This modulation influenced the RPE extracellular matrix deposition, tight junctions and RPE barrier function. In in vivo experiments, the intravitreal administration of recombinant HBEGF (r-HBEGF) alleviated the RPE barrier disruption induced by subretinal injection (SI) or laser treatment and also rescued RPE barrier disruption in endothelial Notch-deficient mice. Our results showed that the endothelial Notch signaling drove HBEGF expression through angiocrine signaling and effectively improved RPE barrier function by regulating the MMP-9 expression in RPE cells. It suggests that the modulation of Notch signaling in the choroidal endothelium may offer a novel therapeutic strategy for retinal degenerative diseases.

## 1. Introduction

The outer blood–retinal barrier (oBRB) is essential for establishing and maintaining optimal retinal function. It comprises the retinal pigment epithelial (RPE) cell layer, Bruch’s membrane, and a rich choroidal vascular plexus [1]. The disturbance or breakdown of the oBRB is a major pathologic trigger for various retinal disorders [2], such as age-related macular degeneration (AMD), diabetic retinopathy (DR), and retinitis pigmentosa (RP). These retinal diseases can cause visual impairment or even blindness, significantly impacting patients’ quality of life [3,4,5]. Previous studies have shown that various factors, such as hypoxia [6], inflammation [7], aging [8], oxidative stress [9], and abnormal extracellular matrix (ECM) deposition [10] are involved in oBRB dysfunction. Thus, restoring oBRB homeostasis is considered an effective strategy for treating several retinal diseases, including oxidative ocular injury.

The main structural feature of oBRB is a monolayer of polarized RPE cells [11], which form a barrier through tight junctions. The RPE cells are positioned between the Bruch membrane and the photoreceptors. They perform various tasks, including light absorption, epithelial transport, spatial ion buffering, the visual cycle, phagocytosis, secretion, and immunological regulation [12]. The hallmark of oBRB establishment during retinal development is the forming of a mature RPE barrier [11]. Underneath the RPE lies the choroid microvascular network, essential for maintaining the RPE barrier. This network consists of choriocapillaris with fenestrated endothelial cells (ECs) and is responsible for supplying nutrients and removing waste from the outer retinal layers [13,14,15]. Notably, genetic and biochemical studies have shown that ECs are a fertile, instructive niche that plays crucial roles in sustaining homeostasis and directing organ regeneration in a “perfusion-independent” manner [16]. Importantly, recent studies have underscored the significance of tissue-specific EC angiocrine signaling in organ growth and renewal, such as liver sinusoidal ECs provoking hepatic proliferation through release of angiocrine signaling factors Wnt2 and hepatocyte growth factor (HGF) [17] and pulmonary ECs initiating regenerative lung alveolarization through angiocrine factor MMP14 [18]. The distinctive spatial structure composition of the RPE and choroidal layers suggests that choroidal EC angiocrine functions may regulate RPE barrier formation. Studies have demonstrated that EC could regulate the RPE barrier function through angiocrine factors [19,20]. However, the specific angiocrine molecules and signaling pathways involved in this process need further clarification.

Notch signaling is a highly conserved developmental pathway in mammals essential for development and tissue renewal. It regulates differentiation, proliferation, and apoptotic cell fates in different cellular contexts [21]. In recent years, there has been increasing attention on the regulatory role of Notch signaling in the tissue-specific angiocrine function of endothelial cells [22]. It was found that endothelial Notch signaling regulated the secretion of Noggin, an antagonist of bone morphogenetic proteins (BMPs), and promoted osteogenesis in bones [22]. Furthermore, our recent research has shown that endothelial-specific activation of Notch signaling disrupted liver regeneration by triggering a senescence-associated secretory phenotype in liver sinusoidal endothelial cells [23]. It obvious that the angiocrine function of EC is specific to organs or tissues, as there are remarkable morphological and functional differences in the vascular beds among different organs [24]. We have previously reported that Notch signaling could regulate multiple functions of EC and play a critical role in maintaining systemic vascular homeostasis in adult mice [25]. Furthermore, we have identified that endothelial-specific activation of Notch signaling inhibits retinal development and intraocular neovascularization in mice [26,27]. However, whether the regulation of the RPE barrier function by choroidal EC angiocrine molecules also depends on Notch signaling remains uninvestigated.

In this study, we observed increased activation of Notch signaling in the choroidal EC of mature oBRB during mouse development. We found that endothelial Notch signaling stimulated the expression of the heparin-binding epidermal growth factor (HBEGF) of ECs through angiocrine signaling. HBEGF is a member of the epidermal growth factor (EGF) family of ligands [28]. It regulates the proliferation of a variety of cells through autocrine or paracrine and plays an important role in a number of pathological and physiological processes including tissue injury and wound healing, angiogenesis, adipogenesis, reproduction, and tumor formation [28,29]. In vitro, exogenous HBEGF stimulation can promote the proliferation and migration of RPE cells [30]. It is also associated with the expression of matrix metalloproteinases (MMPs) [31]. Using a non-contact cell co-culture system, we demonstrated that endothelial Notch signaling stimulated the expression of HBEGF through angiocrine signaling and effectively ameliorated the RPE barrier function by regulating MMP-9 expression in RPE cells. The activation of EC-specific Notch signaling or the intra-vitreous injection of recombinant HBEGF alleviated laser-induced and SI-induced RPE barrier dysfunction. Our finding suggests that modulating Notch signaling in the choroidal endothelium provides a novel therapeutic strategy for retinal degenerative diseases.

## 2. Materials and Methods

### 2.1. Animals

The Animal Experiment Administration Committee of the Fourth Military Medical University in Xi’an, China, approved all animal studies. Mice (C57BL/6J background) were maintained in specific pathogen-free conditions with a 12 h light/dark cycle. The CDH5-CreERT mice, NICD-overexpressing mice (NICD mice), and RBPJ^flox/flox^ mice were generously provided by professor Hua Han from the Air Force Military Medical University. The CDH5-CreERT mice were crossed with the NICD mice and RBPJ^flox/flox^ mice and littermates were genotyped via PCR to obtain CDH5-CreERT-NICD(NICD) mice and CDH5-CreERT-RBPJ^flox/flox^ (RBPJ^f/f^) mice. The Cre recombinase activity of transgenic mice was induced by intraperitoneal injection of tamoxifen (Sigma-Aldrich, Saint Louis, MO, USA). Newborn mice received a total of four injections of tamoxifen (2.5 μL once, 20 mg/mL) from postnatal day 4 (p4) to day 8 (p8). Adult mice (6 weeks old) were injected with five tamoxifen (100 mg/kg) injections. Following the tamoxifen injections, mice were kept for an additional week before undergoing further analyses.

### 2.2. Laser-Induced CNV (Choroidal Neovascularization) Model in Mice

Adult mice (6 weeks old) were anesthetized via intraperitoneal injection of 1% sodium pentobarbital (200 μL/25 g) and 0.5% tropicamide, administered as eye drops to dilate the pupils. Laser photocoagulation (532 nm laser, 200 mW, 100 μm spot size) was performed around the optic disc, avoiding blood vessels. Five laser spots were placed in each eye. For some cases, the CDH5-CreERT-RBPJ^flox/flox^ mice were injected intravitreally with r-HBEGF (2 μL per eye) or IgG after laser induction. The CNV volume was measured 7 days after the procedure. The experiment was performed in at least three biological replicates and at least three animals were used for analysis of each independent experiment.

### 2.3. Sodium Iodate Model in Mice

To induce dysfunction in the RPE barrier, 4-week-old mice were intraperitoneally injected with 30 mg/kg of sodium iodate (SI, Macklin, Shanghai, China) that dissolved in PBS once every 24 h for a total of 5 consecutive days (from P25 to P30) and with PBS as a control. In some cases, the CDH5-CreERT-RBPJ^flox/flox^ mice were injected intravitreally with r-HBEGF (2 μL per eye) or IgG after the SI injection. Mice were sacrificed 5 days after the procedure for analysis and the experiment was performed in at least three biological replicates.

### 2.4. Cell Culture and Treatment

Human umbilical vein endothelial cells (HUVECs, KY20223058-1) were cultured in ECM (ScienCell, Carlsbad, CA, USA) supplemented with 5% fetal bovine serum, 1% endothelial cell growth supplement, and 1% penicillin/streptomycin. The human retinal pigment epithelial ARPE-19 cell lines (RPE) were obtained from the Eye Institute of PLA (iCellbioscience, Shanghai, China). RPE cells were cultured in DMEM/F12 (Hyclone, Logan, UT, USA) medium supplemented with 10% FBS (InCellGene, San Antonio, TX, USA), and 1% penicillin/streptomycin (InCellGene, San Antonio, TX, USA). The primary mouse choroid endothelial cells (CECs) were isolated freshly as described [19] and cultured with advanced DMEM/F12 (Gibco, New York, NY, USA) medium supplemented with 1% ECCG (ScienCell), 20% FBS (InCellGene, San Antonio, TX, USA), antibiotic antifungal 100×-solution (Invitrogen, Waltham, MA, USA), 10 mM HEPES (Invitrogen), 5 μM SB431542 (R&D, Minneapolis, MN, USA), 50 μg/mL heparin (MCE, Belleville, NJ, USA), Glutamax 100×-solution (Gibco, New York, NY, USA), MEM non-essential amino acids 100×-solution (Gibco, New York, NY, USA), 20 ng/mL FGF-2, and 10 ng/mL VEGF (Peprotech, Cranbury, NJ, USA). All cells were cultured at 37 °C in a humidified incubator with 5% CO_2_ and normal oxygen for normoxia treatment, with 5% CO_2_ and 1% O_2_ for hypoxic treatment.

HUVECs were treated with γ-Secretase inhibitor (DAPT, 25 μM, Selleck, Houston TX, USA) when the cells reached 80% confluence for Notch signaling inhibition treatment. For the activation of Notch signaling treatment, HUVECs were infected with NICD overexpressing adenovirus (MOI = 50) when the cells reached 60% confluence. For siRNA transfection treatment, HUVECs were transfected with si-HBEGF (50 nM) using PolyFast transfection reagent (MCE, Belleville, NJ, USA) when the cells reached 80% confluence. After 48 h of treatment, the medium was changed to a typical medium for further analyses or changed to DMEM/F12 medium with 1% FBS for another 48 h to collect conditioned media. The conditioned media were centrifuged (1200 rpm, 5 min) and stored in working aliquots at −80 °C until further use. For recombinant protein stimulation treatment, the r-HBEGF was supplemented at 50 ng/mL in conditioned media before some of the coculture experiments cases, and replaced every 3 days. Experiments were performed in at least three biological replicates per condition.

### 2.5. RPE and HUVEC Cells’ Coculture

A transwell-coculture system was used for the non-contact cell coculture experiment. The ARPE-19 cells were seeded into the polyester transwell inserts (12 mm in diameter, 0.4 mm pore; Corning, Corning, NY, USA) at a density of 5.0 × 10^4^ cells/well. The HUVECs were seeded into 12-well plates (Corning) at a density of 1.0 × 10^5^ cells/well and intervened with the Notch signaling as described before. After the ARPE-19 cells reached 100% confluence, the transwell inserts were moved into the 12-wells with HUVECs and the medium changed to DMEM/F12 with 1% FBS. The cells were maintained under low-serum non-contact coculture conditions for more than 2 weeks for further analyses. For hypoxic treatment, the coculture system was cultured in a 37 °C incubator with 5% CO_2_ and normal oxygen for 14 days and then transferred to a 37 °C incubator with 5% CO_2_ and 1% O_2_ for 3 days. Experiments were performed in at least three biological replicates per condition.

### 2.6. RPE Polarization and TER Measurements

The ARPE-19 cells were seeded into the transwell inserts and cultured for 21 days for RPE cell polarization. Then, the transepithelial resistance (TER) was measured using an EVOM voltohmmeter (World Precision Instruments, Sarasota, FL, USA) every three days during the cell culture period, and the value of a non-cultured cell well was used as the background resistance. The resistance value was measured 8 times per well and the average values were expressed in ohms (O) cm^2^ after background subtraction. When the TER value was stable for three consecutive days, the RPE barrier was considered to be formed. Experiments were performed in at least three biological replicates per condition.

### 2.7. Immunofluorescence Assays

To assess RPE cell polarization, ARPE-19 cells on the transwell insert membrane were fixed with 4% paraformaldehyde (PFA) for 15 min at RT. For the extracellular matrix protein deposition assay of the RPE cells, transwell inserts were washed with PBS (InCellGene, San Antonio, TX, USA) and then incubated in 0.02 M NH_4_OH (Sigma-Aldrich, Saint Louis, MO, USA) for 10 min to remove adherent cells. This was followed by fixing the extracellular matrix protein with 4% paraformaldehyde (PFA) for 15 min at RT. We removed the insert membrane from the transwell with a blade and placed in a 12-well plate. Then, we blocked the cells on the insert membrane with PBS containing 0.5% Triton X-100 (Sigma-Aldrich, Saint Louis, MO, USA) and 3% BSA (Sigma-Aldrich, Saint Louis, MO, USA) for 45 min at RT. For choroid whole flat mount staining, eyes were enucleated and fixed with 4% paraformaldehyde (PFA) for 2 h at RT. After removing the anterior segment and neural retina using a dissecting stereomicroscope, the choroid/sclera complex was blocked with PBS containing 0.5% Triton X-100 and 1% BSA overnight at 4 °C. After the block, the samples (the inserts or choroid/RPE complexes) were rinsed with PBS at RT and incubated with primary antibodies to zonula occludens-1 (ZO-1; 1:200, ab221547, Abcam, Cambridge, UK), fibronectin (1:200, ab268020, Abcam, Cambridge, UK), collagen IV (1:50, sc-59814, Santa Cruz, Dallas, TX, USA), and isolectin-B4 (IB4,1:100, FL-1201, Vectorlabs, Burlingame, CA, USA) overnight at 4 °C. The next day, we removed the primary antibody and washed the samples three times with PBS at RT. Then, we incubated the samples with fluorescence-conjugated secondary antibody (Invitrogen, Alexa Fluor 488-goat anti-rabbit, Alexa Fluor 594-goat anti-mouse) for 2 h at RT in the dark. We removed the secondary antibody and washed the samples three times with PBS. For the RPE polarization assay, the insert membranes also needed to be incubated with DAPI (Invitrogen) for 10 min to stain the nuclei. Finally, we placed the samples carefully on a glass slide and gently pressed to leave them as flat as possible. The samples were visualized using a laser-scanning confocal fluorescence microscope (Nikon A1R, Tokyo, Japan).

For CNV volumetric analysis, Z-stack images of CNV spots were processed using the imaris software (verision:7.7.1)and the neovascular volume was measured and compared. At least three mice were used for each different treatments.

The primary antibodies used in immunofluorescence assays are shown in Table 1.

### 2.8. HBEGF Secretion Assays

The cells were treated to inhibit or activate the Notch signaling in HUVECs for 48 h, as described before and collection in the culture media. Then, the concentration of secreted HBEGF in the media was measured using the human HBEGF ELISA kit (Boster, Wuhan, China). Experiments were performed in at least three biological replicates per condition.

### 2.9. MMP-9 Enzyme Activity Assay

We collected the media from the RPE-HUVEC non-contact coculture system as described before and concentrated it 30-fold using an ultrafiltration tube with a 10 kDa molecular weight cutoff (Pall Life Sciences, Covina, CA, USA). Enzyme activity was measured using the MMP gelatin assay kit (Shanghai Jiemei Gene Medicine Technology Co., Ltd., Shanghai, China). ImageJ software (version 1.49p; National Institutes of Health) was used for measuring and analyzing the density. Experiments were performed in at least three biological replicates per condition.

### 2.10. RT- qPCR Assays

Total RNA was lysed in a reagent (RNAiso Plus, Takara, Japan) and reverse transcribed into complementary DNA (cDNA) using a reverse transcription kit (PrimeScript™ RT Master mix, Takara, Osaka, Japan). The StepOne Plus Real Time PCR System (Life Technologies, Carlsbad, CA, USA) was used along with a PCR kit (TB Green^®^ Premix Ex Taq™ II; Takara, Shiga, Japan) to perform real-time quantitative RT-PCR analyses. The primer sequences used in this experiment are shown in Table 2.

### 2.11. Western Blot Assays

Cells were lysed with RIPA lysis buffer (Beyotime, Nanjing, China) supplemented with a protease inhibitor cocktail (1697498, Roche, Basel, Switzerland) and protein concentration was measured with the Pierce bicinchoninic acid (BCA) assay kit (Beyotime, Nanjing, China). An equal amount of protein was separated by 8% or 10% sodium dodecyl sulfate-polyacrylamide gel electrophoresis and transferred onto a polyvinylidene fluoride (PVDF) membrane. After being blocked with PBST containing 5% skim milk for 2 h, the membranes incubated overnight at 4 °C in primary antibodies: against ZO-1 (1:1000, 13663S, CST, Danvers, MA, USA), HBEGF (1:1000, ab185555, Abcam, Cambridge, UK), MMP-9 (1:1000, ab76003, Abcam, Cambridge, UK), anti-β-tubulin (1:1000, 2148, CST, Danvers, MA, USA), and β-actin (1:1000, ab5316, Abcam, Cambridge, UK). The membranes were washed and incubated with secondary antibodies: horseradish peroxidase (HRP)-linked goat anti-rabbit IgG (1:1000, SA00001-2, Proteintech, Wuhan, China) and horse anti-mouse IgG (1:1000, 7074, Cell Signaling Technology, Danvers, MA, USA) antibodies for 2 h at RT. After washing with PBST solution, the bands were detected via enhanced chemiluminescence (ECL; ZETA, Beijing, China). ImageJ software (version 1.49p; National Institutes of Health, Bethesda, MD, USA) was used for measuring and analyzing the density ratio of the target proteins relative to the internal reference protein (β-actin or β-Tubulin). Three biological replicates were performed for each protein. The primary antibodies used in Western blotting assays are shown in Table 3.

### 2.12. Statistics

Statistical analysis was performed using GraphPad Prism 9 software. All quantitative data is expressed as mean ± SEM. Statistical significance was determined via one-way variance analysis (ANOVA) and unpaired or paired Student’s *t*-test. A *p*-value of <0.05 was considered statistically significant.

## 3. Results

### 3.1. Inhibiting Notch Signaling in Choroid Endothelial Cells (CECs) Affects the RPE Barrier Formation and Aggravates the RPE Barrier Disruption Induced by Sodium Iodate

**Figure 1:**
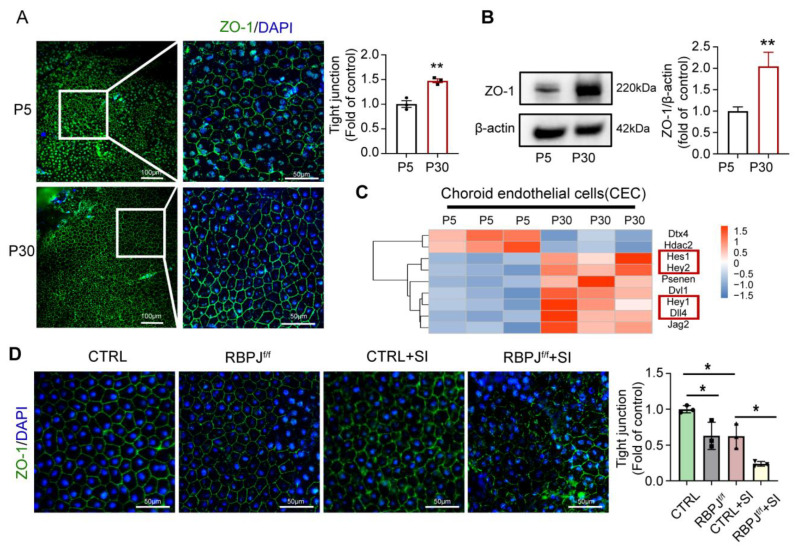
Notch signaling in CECs regulates the RPE barrier formation and the disruption induced by sodium iodate. (**A**) Whole-mount choroidal staining with ZO-1 in 5-day-old and 30-dayold mice. The number of RPEs with intact tight junctions was quantitatively compared. Scale bars: 100 µm, 50 µm. (**B**) Protein expression level of ZO-1 in choroidal issue of P5 and P30 mice was tested via Western blot. (**C**) A heat map of differential gene expression in the CECs of P5 and P30 mice. Each row in the heat map represents a differential gene, each column represents a sample, and the color represents the expression level. Blue to red corresponds to the expression level, from low to high. (The red box are genes associated with the Notch signal) (**D**) Whole-mount choroidal staining of EC-specific Notch signaling deletion mice CDH5-CRE-RBPJ^f/f^ (RBPJ^f/f^) or control (RBPJ^f/+^) mice at p30, treated with or without SI, were analyzed using ZO-1 staining. The number of RPEs with intact tight junctions was quantitatively compared. Scale bars: 50 µm. * *p* < 0.05, ** *p* < 0.01. Three biological replicates were performed and values presented as the mean ± SEM (*n* = 3). * *p* < 0.05, ** *p* < 0.01.

We first observed the formation of the RPE barrier during retinal development. A monolayer of polarized cells forms the RPE barrier with tight junction proteins like zonula occludens-1 (ZO-1). Whole-mount choroidal staining with ZO-1 at P5 and P30 shows that the relative expression of ZO-1 in the RPE of P30 mice is higher compared with that of P5, and the tight connection of the RPE is more integral at P30 (Figure 1A). According to a previous study [2], a mature choroid microvascular network under the RPE layer contributes to the formation of the RPE barrier. Through gene enrichment analysis on the transcriptome data of CECs in 5-day-old and 30-day-old mice, we noticed that, compared with P5 CECs, P30 CECs express higher levels of the Notch signaling pathway related to molecules such as Hes1, Hey1, Hey2, and Dll4 (Figure 1C). This finding implies that the activation of Notch signaling in ECs may be involved in the formation and maturation of the RPE barrier function. To demonstrate the impact of endothelial Notch signaling in the RPE barrier, we used EC-specific Notch signaling deletion mice CDH5-CRE-RBPJ^f/f^ to inhibit endothelium Notch signaling. Whole-mount choroidal staining with ZO-1 reveals that the tight junctions of RPE cells are weaker in the Notch signaling deletion mice compared to the control mice (Figure 1D). In the sodium iodate (SI)-induced RPE barrier disruption model, we observed that the endothelium Notch signaling inhibition aggravates the disruption of the tight junction of RPE layers (Figure 1C). The findings further confirm that ECs play a crucial role in RPE barrier formation and indicate that Notch signaling in the endothelium is involved in regulation.

### 3.2. Endothelial Notch Signaling Promotes RPE Barrier Formation through Angiocrine Function In Vitro

**Figure 2:**
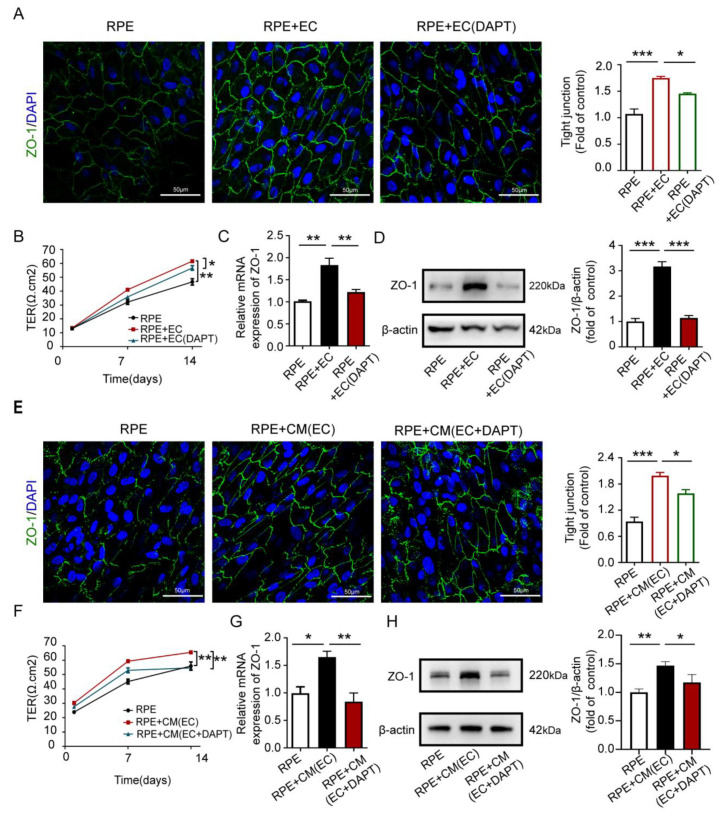
Endothelial Notch signaling promotes RPE barrier formation through angiocrine function in vitro. (**A**) Non-contact coculture model with ECs and ARPE-19 cells in vitro for RPE barrier formation. The tight junctions were analyzed using ZO-1 staining in RPE, RPE with HUVECs, and RPE with Notch signaling-inhibited HUVEC. The number of RPEs with intact tight junctions was quantitatively compared. Scale bars: 50 µm. (**B**) The TER values of RPE barrier were compared in RPE, RPE with HUVECs, and RPE with Notch signaling-inhibited HUVECs. (**C**,**D**) mRNA and protein levels of ZO-1 in RPE cells were tested in RPE, RPE with HUVECs, and RPE with Notch signaling-inhibited HUVECs via qRT-PCR and Western blot. (**E**–**H**) Culture of the RPE with the HUVEC-conditioned medium. The ZO-1 staining, TER values, mRNA, and protein levels of ZO-1 were analyzed as before in RPE cells cultured in normal medium and RPE cells cultured in EC-conditioned medium. Three biological replicates were performed and values are presented as the mean ± SEM (*n* = 3). * *p* < 0.05, ** *p* < 0.01, *** *p* < 0.001.

To further confirm the specific regulation of endothelium Notch signaling on RPE barrier function in vitro, we established a non-contact coculture model with ECs and ARPE-19 cells. For the cell model, the ECs and ARPE-19 cells were seeded in two different compartments; the ARPE-19 cells were seeded into insert membranes and the ECs were seeded into wells, and cells could indirectly communicate through the pores of the membrane. The transepithelial electrical resistance (TER) value and tight-junction protein ZO-1 expression can be used to assess the RPE barrier function. We cultured the RPE cells on polyester transwell inserts until full confluence and transferred them to new cell culture chambers along with ECs or conditioned media. We used HUVECs and primary CECs for coculture experiments and found that both ECs had no significantly different effects on RPE (Supplementary Figure S1). Therefore, we executed coculture experiments using HUVECs. The RPE cells improve tight junction morphology, as seen in the ZO-1 fluorescence staining (Figure 2A), and exhibit higher TER values in the presence of ECs (Figure 2B). Additionally, the expression of ZO-1 in RPE cells upregulated the mRNA and protein levels (Figure 2C,D). However, both the TER values and the ZO-1 expression of the RPE decreased significantly with DATP treatment to inhibit Notch signaling in the ECs. Furthermore, we observed similar results by using an EC-conditioned medium for RPE cell culture (Figure 2E–H). Overall, these findings confirm, in vitro, that coculture with ECs can effectively promote the formation of the RPE barrier through angiocrine function, and this effect is attenuated when endothelial Notch signaling is blocked.

### 3.3. Endothelial Notch Signaling Promotes ECM Deposition in the Formation of the RPE Barrier

**Figure 3:**
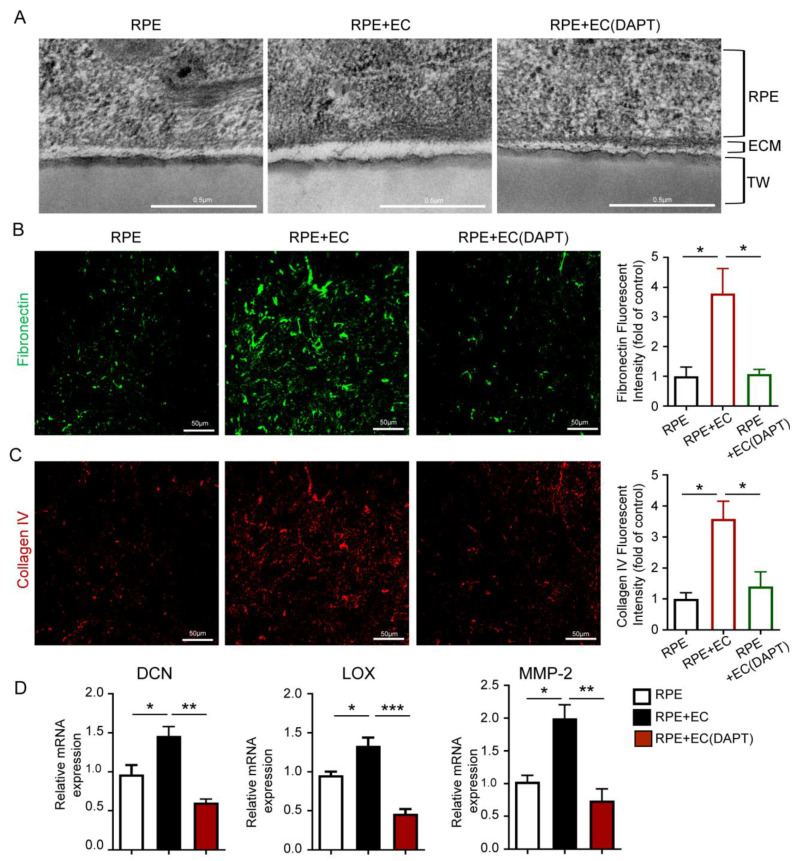
Endothelial Notch signaling promotes ECM deposition in the formation of the RPE barrier. (**A**) The HUVECs and RPE cells were cocultured as before and the ECM layer of the RPE barrier was observed in RPE, RPE with HUVECs, and RPE with Notch signaling-inhibited HUVECs using transmission electron microscopy. Scale bars: 0.5 µm. (**B**,**C**) The ECM deposition protein expressions were analyzed in the RPE, RPE with HUVECs, and RPE with Notch signaling-inhibited HUVECs via fibronectin and collagen IV staining outside the RPE cells. Fibronectin- and collagen IV-positive areas were quantitatively compared. Scale bars: 50 µm. (**D**) mRNA levels of ECM-related genes DCN, LOX, and MMP-2 in RPE cells were tested in the RPE, RPE with HUVEC, and RPE with Notch signaling-inhibited HUVECs via qRT-PCR. Three biological replicates were performed and values are presenten as the mean ± SEM (*n* = 3). * *p* < 0.05, ** *p* < 0.01, *** *p* < 0.001.

It has been reported that the extracellular matrix (ECM) can regulate the function of the RPE barrier [19]. Therefore, we sought to explore whether inhibiting endothelial Notch signaling affects the deposition of ECM in polarized RPE cells. The transmission electron microscopy analysis demonstrates a relatively thin layer of ECM under the polarized RPE cells when the RPE barrier formed. Using the coculture system, we found that the ECM layer is more abundant in the presence of ECs (Figure 3A). Immunofluorescence analyses of the transwell inserts’ membrane show an increased expression of fibronectin and collagen IV proteins in the presence of ECs (Figure 3B,C). RT-qPCR analysis of the RPE cells reveals an upregulation of ECM-related genes, including decorin (DCN), lysyl oxidase (LOX), and MMP-2, in the presence of ECs (Figure 3D). However, when inhibiting the Notch signaling in HUVECs via the DATP treatment, the ECM layer, fibronectin, and collagen IV proteins’ expression, the ECM-related gene’s expression exhibited a similar level to that of the absence of ECs (Figure 3A–C). These results suggest that ECs can promote the deposition of ECM occurring in the RPE barrier through angiocrine regulation via Notch signaling.

### 3.4. Endothelial Notch Signaling Activation Alleviates RPE Barrier Dysfunction in Hypoxia through Angiocrine Signaling

**Figure 4:**
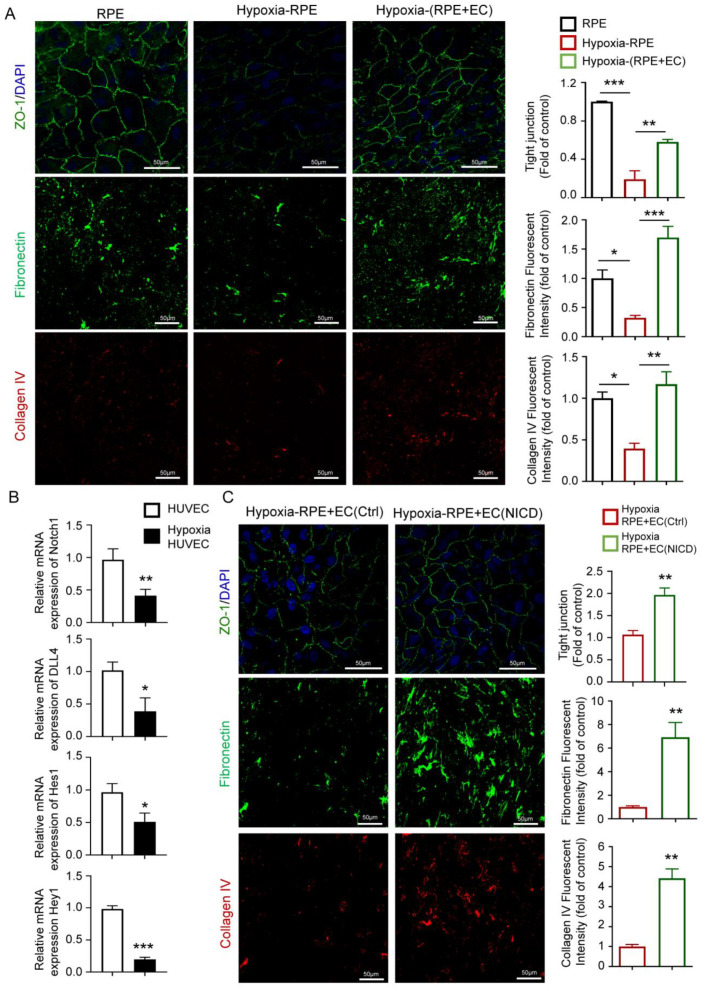
Endothelial Notch signaling activation alleviates RPE barrier dysfunction in hypoxia through angiocrine signaling. (**A**) Evaluating the RPE barrier function of RPE in the normal culture condition, hypoxia culture condition, and hypoxia RPE–EC coculture condition using ZO-1 staining (top), fibronectin staining, (middle) and collagen IV staining (bottom). The number of RPEs with intact tight junctions was quantitatively compared. Fibronectin- and collagen I-positive areas were quantitatively compared. Scale bars: 50 µm. (**B**) mRNA levers of Notch signaling-related genes were tested in HUVECs cultured with normal conditions and hypoxia conditions for 48 h and established via qRT-PCR, including Notch1, Dll4, Hey1, and Hes1. (**C**) Evaluating the RPE barrier function of RPE-HUVECs coculture and RPE-Notch signaling-inhibited HUVECs coculture in hypoxia condition using ZO-1 staining (top), fibronectin staining (middle), and collagen IV staining (bottom). The number of RPEs with intact tight junctions was quantitatively compared. Fibronectin- and collagen I-positive areas were quantitatively compared. Scale bars: 50 µm. Three biological replicates were performed and values are presented as the mean ± SEM (*n* = 3). * *p* < 0.05, ** *p* < 0.01, *** *p* < 0.001.

Hypoxia is one of the common factors inducing RPE barrier dysfunction. It is implicated in the development of retinal degenerations such as retinitis pigmentosa (RP), age-related macular degeneration (AMD), and diabetic retinopathy (DR) [32]. In this study, the culture of RPE cells with HUVECs under hypoxic conditions confirms that hypoxia severely damages the functioning of the RPE barrier, which is evident from the significant decrease in the tight junction of RPE and the expression of ECM-related proteins, collagen IV and fibronectin (Figure 4A). However, we also noticed that ECs partially rescue the RPE barrier dysfunction induced by hypoxia (Figure 4A). Interestingly, RT-qPCR reveals that the Notch signaling-related genes (Notch1, Dll4, Hey1, and Hes1) are downregulated in HUVECs when cultured under hypoxic conditions (Figure 4B). Based on this observation, the activation of Notch signaling in HUVECs must enhance the protective role of ECs on the RPE barrier under hypoxic conditions. Using NICD adenovirus infection on the HUVECs, we continuously activated Notch signaling in ECs and the ECs cocultured with RPE under hypoxic conditions. Notch signaling prompts ECs to improve the RPE barrier function by upregulating the expression of the tight junction protein ZO1 and ECM-related proteins, collagen IV and fibronectin, in RPE cells compared with the control group (Figure 4C). Furthermore, to illustrate the role of activated endothelial Notch signaling in the in vivo model, we used an EC-specific NICD-overexpressing mouse CDH5-CRE-NICD and established a laser-induced CNV model. Compared to control mice, whole-mount staining with IB4 shows that the NICD-overexpressing mice exhibit reduced CNV injury and upregulate the expression of the ECM-related genes collagen IV and lysyl oxidase (LOX) (Supplementary Figure S2). These findings reveal that EC can alleviate the RPE barrier dysfunction caused by hypoxia through secreted factors, and Notch signaling activation enhances this protective effect.

### 3.5. Endothelial Notch Signaling Affects RPE Barrier Function by Regulating the HBEGF Paracrine of ECs

**Figure 5:**
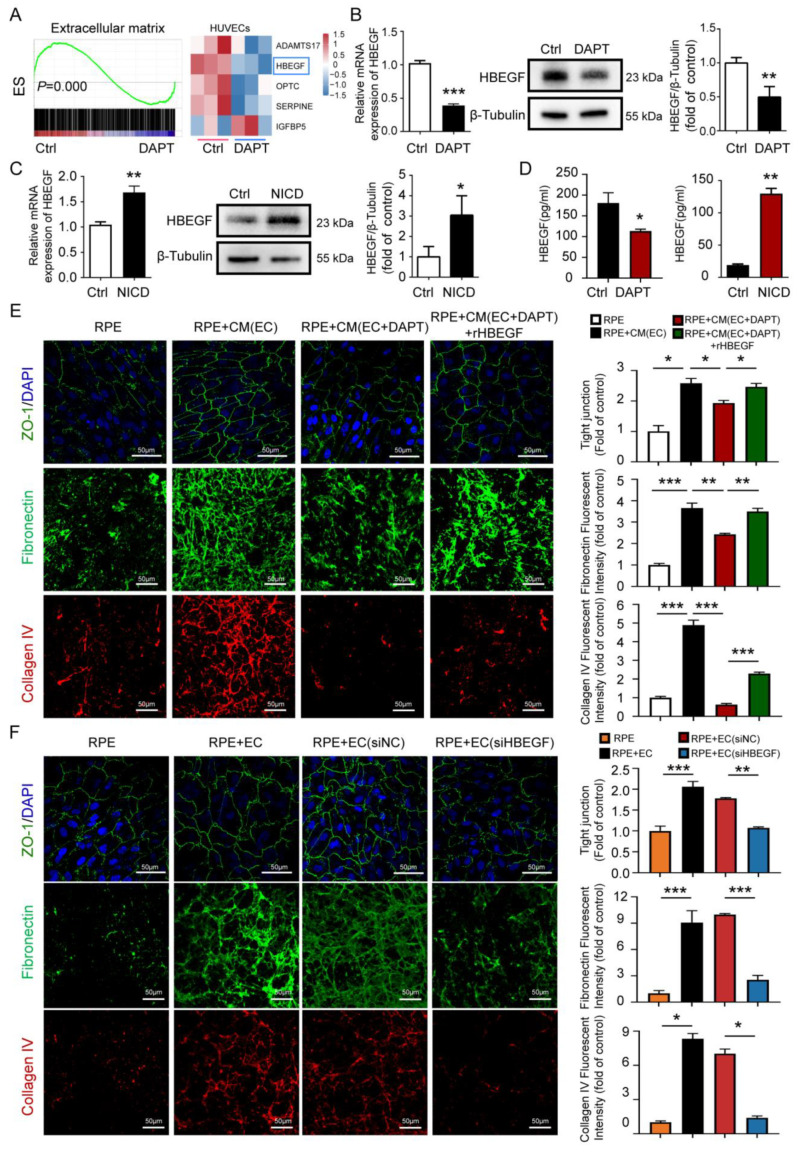
Endothelial Notch signaling affects RPE barrier function by regulating the HBEGF paracrine of EC. (**A**) Analysis for previously published transcriptome sequencing data of normal HUVECs and DAPT treated HUVECs: the GSEA results for ECM (**left**) and heatmap of the expression of ECM-related genes (**right**). (**B**) mRNA and protein levels of HBEGF in normal HUVECs and DAPT-treated HUVECs. (**C**) mRNA and protein levels of HBEGF in normal HUVECs and NICD adenovirus-infected HUVECs. (**D**) The secretion levels of HBEGF protein in normal HUVECs and DAPT-treated HUVECs or NICD adenovirus-infected HUVECs were tested using an ELISA kit. (**E**) Evaluating the RPE barrier function in RPE cells cultured in normal medium, RPE cultured in the EC-conditioned medium, RPE cultured in Notch signaling-inhibited EC-conditioned medium, and RPE cultured in Notch signaling-inhibited EC-conditioned medium supplemented with recombinant HBEGF (r-HBEGF) using ZO-1 staining (top), fibronectin staining (**middle**), and collagen IV staining (bottom). The number of RPEs with intact tight junctions were quantitatively compared. Fibronectin- and collagen IV-positive areas were quantitatively compared. Scale bars: 50 µm. (**F**) Evaluating the RPE barrier function in the RPE, RPE with HUVECs, RPE with siHBEGF-treated HUVECs, and RPE with siNC-treated HUVECs using ZO-1 staining (top), fibronectin staining (**middle**), and collagen IV staining (bottom). The number of RPEs with intact tight junctions were quantitatively compared. Fibronectin- and collagen IV-positive areas were quantitatively compared. Scale bars: 50 µm. Three biological replicates were performed and values are presented as the mean ± SEM (*n* = 3). * *p* < 0.05, ** *p* < 0.01, *** *p* < 0.001.

To determine the critical paracrine factors of ECs that might regulate the RPE barrier, we next reanalyzed previously published transcriptome sequencing data of HUVECs treated with DAPT. The gene set enrichment analyses revealed a lower enrichment of ECM-related genes in the group of ECs for which Notch signaling is blocked (Figure 5A). Specifically, the paracrine factor HBEGF significantly decreases when Notch signaling is blocked in ECs (Figure 5A). We then confirmed the sequencing data at the transcriptional and translational levels. Both mRNA expression levels and HBEGF protein secretory levels are down-regulated in Notch signaling-deficient ECs (Figure 5B,D). Accordingly, when Notch signaling is activated constructively in ECs, the HBEGF expression increased (Figure 5C,D). To further investigate whether HBEGF functions in the endothelium Notch blockade induced the RPE barrier function disruption, we added recombinant HBEGF (r-HBEGF) protein into EC-conditioned medium. The tight junction of RPE (stained with ZO-1) and the deposits of collagen IV and fibronectin outside the RPE significantly increased after the addition of recombinant protein HBEGF compared to the Notch signaling-deficient EC-conditioned medium with the control group (Figure 5E). Conversely, the si-HBEGF-treated HUVECs-conditioned medium attenuates the protective function of the ECs on the RPE barrier (Figure 5F). These results indicate that endothelial Notch signaling affects RPE barrier function by regulating the HBEGF paracrine signaling of ECs.

### 3.6. r-HBEGF Ameliorated the RPE Barrier Dysfunction of EC-Specific Notch Signaling Deletion Mice

**Figure 6:**
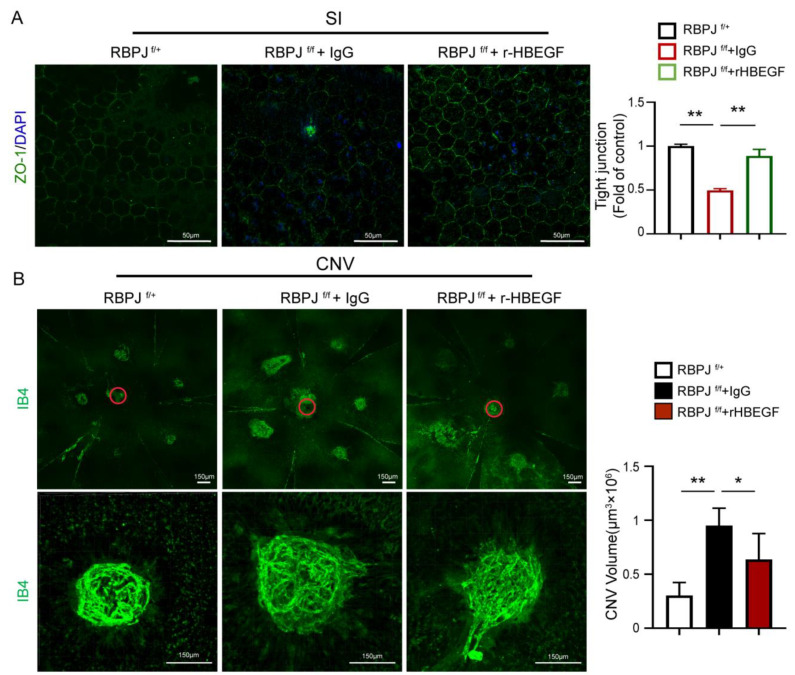
r-HBEGF improved the RPE barrier dysfunction of Notch signaling deletion mice. (**A**) In the SI-induced RPE barrier disfunction model, whole-mount choroidal staining for EC-specific Notch signaling deletion mice CDH5-CRE-RBPJ^f/f^ intravitreally injected with IgG or r-HBEGF, and control mice (RBPJ^f/+^) at p30, were analyzed using ZO-1 staining. The number of RPE cells with intact tight junctions were quantitatively compared. Red circles indicate the location of the optic disk. Scale bars: 50 µm. (**B**) In the laser-induced CNV model, whole-mount choroidal staining for EC-specific Notch signaling deletion mice CDH5-CRE-RBPJ^f/f^ intravitreally injected with IgG or r-HBEGF and control mice (RBPJ^f/+^) were analyzed using IB-4 staining. IB4 positive areas were quantitatively compared. Three biological replicates were performed and values are presented as the mean ± SEM (*n* = 3). Scale bars: 150 µm. * *p* < 0.05, ** *p* < 0.01.

Next, we assessed the therapeutic possibility of r-HBEGF improving RPE barrier dysfunction via intravitreal injection in vivo. We established the SI-induced RPE barrier dysfunction model and laser-induced CNV model in EC-specific Notch signaling deletion mice CDH5-CRE-RBPJ^f/f^. As previously mentioned, the dysfunction of the REP barrier is more severe in CDH5-CRE-RBPJ^f/f^ mice after SI induction. We injected r-HBEGF or IgG 1 day after SI induction and harvested the eyeballs after P5. Whole-mount choroidal staining with ZO-1 shows that injection of the r-HBEGF protein rescued the RPE barrier disruption induced by SI (Figure 6A). Similar effects were also observed in the CNV model, where r-HBEGF or IgG was injected 1 day and 3 days after laser induction in adult CNV model mice and choroidal tissues were collected after 7 days. Whole-mount choroidal staining with IB4 shows that the injection of r-HBEGF rescued the CNV injury in CDH5-CRE-RBPJ^f/f^ mice (Figure 6B). Consequently, these findings demonstrate that the r-HBEGF protein can partially ameliorate the RPE barrier dysfunction induced by SI and lasers in vivo, and these findings also suggest that the r-HBEGF protein has the potential to be a novel interventional therapeutic strategy for improving RPE barrier dysfunctions related to diseases.

### 3.7. Notch Signaling-Driven Endothelial HBEGF Improved RPE Barrier Function by Regulating MMP-9 Expression in RPE Cells

**Figure 7:**
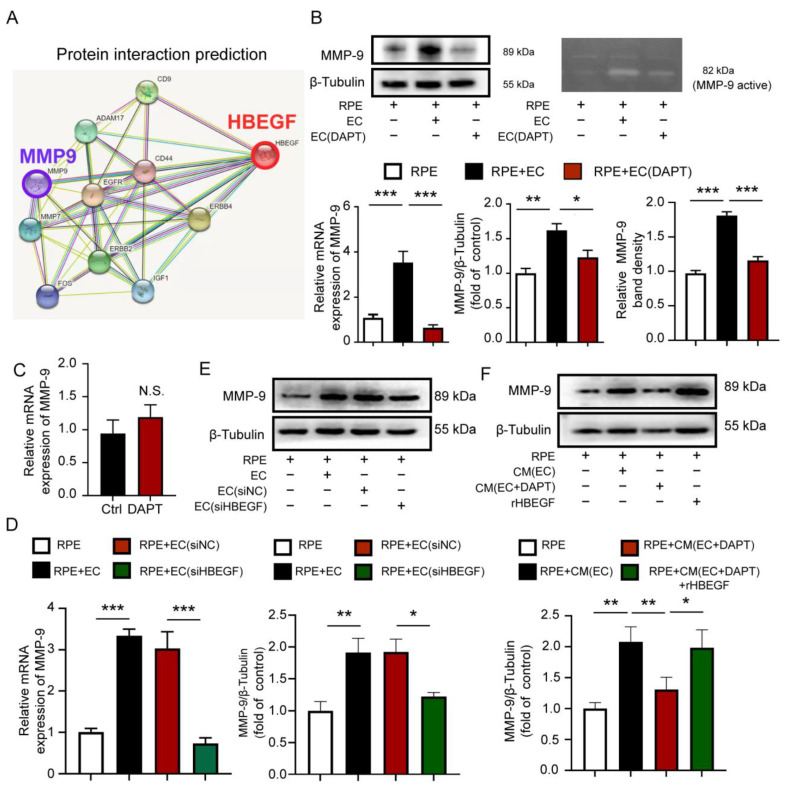
EC regulates MMP-9 expression in RPE cells via Endothelial Notch signaling-driven HBEGF secretion. (**A**) Protein interaction prediction analysis results of HBEGF and MMP-9, using the String database. (**B**) mRNA level, protein level, and enzyme activity level of MMP-9 in the RPE, RPE with HUVEC, and RPE with Notch signaling-inhibited HUVECs were tested via qRT-PCR, Western blot, and gelatin assay kit. (**C**) mRNA levels of MMP-9 in normal HUVECs and DAPT-treated HUVECs were tested via qRT-PCR. (**D**,**E**) mRNA and protein levels of MMP-9 in the RPE, RPE with HUVECs, RPE with siNC-treated HUVECs, and RPE with siHBEGF-treated HUVECs were tested via qRT-PCR and Western blot. (**F**) Protein levels of MMP-9 in RPE cells cultured in normal medium, RPE cultured in EC-conditioned medium, RPE cultured in Notch signaling-inhibited EC-conditioned medium, and RPE cultured in Notch signaling-inhibited EC-conditioned medium supplemented with recombinant HBEGF (r-HBEGF) were tested using Western blot. Three biological replicates were performed and values are presented as the mean ± SEM (*n* = 3). NS, no significance, * *p* < 0.05, ** *p* < 0.01, *** *p* < 0.001.

**Figure 8:**
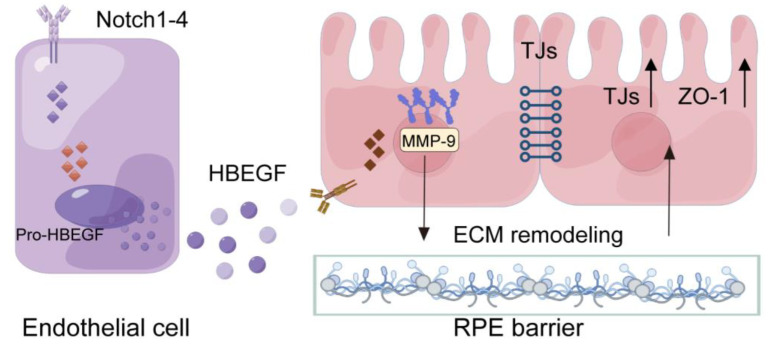
Scheme of endothelial Notch signaling regulating the RPE barrier function.

Protein interaction prediction analysis using the String database reveals a potential protein interaction between HBEGF and MMP-9, a critical enzyme that participates in ECM remodeling (Figure 7A). To confirm that, we detected the MMP-9 expression of RPE cells in the presence of HUVECs. We found that it was upregulated at both the mRNA (RT-qPCR) and protein (Western blot) levels (Figure 7B). Furthermore, the enzyme activity assay conducted in the supernatant of cultured RPE cells shows that the activation of MMP-9 increased in the presence of ECs (Figure 7B). However, the inhibition of Notch signaling in ECs via DAPT treatment or the inhibition of HBEGF expression in ECs by si-HBEGF effectively reduces the Notch/HBEGF-elicited upregulation of MMP-9 in RPE cells, and increases the MMP-9 activity in the supernatant (Figure 7B,D,E). Moreover, the expression of MMP-9 in ECs does not show any significant difference with DAPT treatment (Figure 7C), implying that the increase in activated MMP-9 is produced mainly by RPE cells. To further verify the interaction between HBEGF and MMP-9 in the RPE, we added HBEGF recombinant protein in the Notch signaling-blocked HUVECs-conditioned medium to the RPE culture system. The result shows that the downregulation of MMP-9 expression was rescued with the addition of r-HBEGF (Figure 7F). These findings imply that endothelial Notch signaling drives HBEGF through angiocrine signaling and effectively improves the barrier function of the RPE by regulating MMP-9 expression in RPE cells (Figure 8). 

## 4. Discussion

The oBRB dysfunction caused by RPE barrier breakdown is a significant factor in various retinal diseases, severely affecting visual function. Therefore, an in-depth understanding of the mechanisms that regulate the RPE barrier function is viral. Tissue-specific ECs modulate tissue homeostasis and regenerative processes [16]. In this study, we focused on the role and molecular mechanisms of choroidal tissue-specific ECs in regulating and maintaining the RPE barrier function. We find choroidal EC angiocrine signaling during retinal development, which is involved in regulating the formation and maintenance of the RPE barrier by enhancing the tight junctions of RPE and promoting the deposition of ECM under the RPE layer, which is consistent with the findings of Benedicto et al. [19]. Interestingly, by comparing the transcriptome data of choroidal EC before and after visual maturation, we notice that the Notch signaling in choroidal EC might be involved in the formation of the RPE barrier function. Previous studies have demonstrated that the Notch signaling pathway regulates various EC behaviors involved in angiogenesis and vascular remodeling, including proliferation, adhesion and migration [21]. Furthermore, it revealed that this pathway also maintains organ homeostasis through EC angiocrine signaling [33,34]. For instance, in bone, Notch signaling promoted the bone vasculature angiocrine platform, harmonizing bone formation and regeneration [35]. In the liver, sustained Notch signaling in the liver endothelium led to angiocrine landscape changes and increased liver fibrosis upon damage [36]. In the brain, EC Notch signaling suppressed caveolae formation and subsequent pathological transcytosis across the blood–brain barrier (BBB) in adults [37]. Here, by using EC-specific Notch signaling deletion mice and DAPT in vitro to inhibit endothelium Notch signaling, we first confirm that endothelium Notch signaling regulates the formation of the RPE barrier by modulating EC angiocrine signaling. Our result further enriches the regulatory function of Notch signaling on tissue-specific endothelium.

Hypoxia is a common factor that induces dysfunction of the RPE barrier in developing retinal diseases, such as diabetes and AMD [38]. Hypoxia disrupts the tight junctions between RPE cells, resulting in increased cell permeability, it induces inflammation, activates oxidative stress, and ultimately leads to oBRB catabolism [39,40]. The choroidal EC function is also abnormal in retinal diseases with a disrupted RPE barrier. Using non-contact cell co-culture experiments, we find that the hypoxic choroidal ECs retain their protective role in the RPE barrier. Notably, the sustained expression of Notch signaling in ECs by NICD adenoviral infection led Notch-activated ECs to improve RPE barrier function by angiocrine signaling under hypoxic insult. Further in vivo analysis confirms that EC-specific Notch activation attenuates the RPE barrier dysfunction in the laser-induced CNV model in mice. These findings suggest that activating EC Notch signaling is a potential therapeutic strategy to restore the RPE barrier. In the onset of retinal diseases, other factors such as oxidative stress, high glucose, toxicity, and inflammation also cause RPE barrier dysfunction. Similarly, in the SI-induced RPE barrier disruption model, we observe that inhibition of the activation of EC Notch signaling aggravates the disruption of the RPE barrier. Thus, our studies are the first to report that endothelial Notch signaling can regulate angiocrine signaling and is involved in regulating the oBRB function.

What are the underlying mechanisms? Consistent with previous studies [19,20,41], we confirm that EC promotes the deposition of the ECM layer under the RPE layer through EC angiocrine signaling [19,20,41]. The ECM, which contains collagen, fibronectin, elastin, laminin [42,43], and matrix metalloproteinase [44], provides structural support for the RPE layer in oBRB [45]. Benedicto et al. identified that EC-secreted lysyl oxidase remodeled the ECM and enhanced RPE barrier function [19]. Until now, the critical paracrine factor of CECs which regulates the RPE barrier function was poorly understood. Based on the transcriptome sequencing data analysis of Notch signaling blocking ECs, this study focuses on HBEGF, a paracrine factor of EC that is regulated by Notch signaling. It confirms that endothelial Notch signaling affects RPE barrier function by regulating the HBEGF paracrine of EC. In addition, we find that endothelial Notch signaling-driven HBEGF secretion regulates the expression and activity of the ECM-related enzyme MMP-9 in RPE cells. This rationale is also supported by a previous study conducted on the blood–brain barrier [31]. Further analysis confirms that the recombinant HBEGF protein partially ameliorates the RPE barrier dysfunction induced by SI and lasers in vivo. However, it remains to be explored whether other secreted factors regulated by EC Notch signaling are also involved in the protective function of EC against RPE barrier dysfunction, and this deserves future investigation.

In conclusion, this study supports a novel mechanism of EC-RPE crosstalk in regulating the outer blood–retinal barrier (oBRB) function. Endothelial Notch signaling regulates choroid angiocrine signaling, while RPE cells sense the stimulation of the secretory factor HBEGF. This leads to an up-regulation of MMP-9 expression and activity, initiating extracellular matrix (ECM) remodeling, enhancing the tight junctions between RPE cells, and ultimately protecting the function of the oBRB. This study only briefly explored the interactions between HBEGF and MMP-9. Further investigations are needed to examine the protein binding sites and activation mechanisms of HBEGF and MMP-9 in depth. These results expand our understanding of the role of endothelial Notch signaling in tissue-specific endothelial angiocrine regulation. They imply that modulating Notch signaling in the choroidal endothelium may provide a novel therapeutic strategy in retinal degeneration diseases.

## Figures and Tables

**Table 1:** Primary antibodies used in immunofluorescence assays.

Antibody	Concentration	Catalogue Number	Company
ZO-1	1:200	ab221547	Abcam
Fibronectin	1:200	ab268020	Abcam
CollagenIV	1:500	sc-59814	Santa Cruz
isolectin-B4	1:100	FL-1201	Vectorlabs

**Table 2:** Primer sequences used in this study.

Gene Name	Species	Gene Accession Number	Primers	Primer Sequence 5′→3′	Product Length (bp)
*β-actin*	*human*	NM_001101.5	Forward	CTCCATCCTGGCCTCGCTGT	268
			Reverse	GCTGTCACCTTCACCGTTCC	
*ZO-1*	*human*	NM_001301025.3	Forward	ACCAGTAAGTCGTCCTGATCC	128
			Reverse	TCGGCCAAATCTTCTCACTCC	
*Hey1*	*human*	NM_001040708.2	Forward	GAGTGCGGACGAGAATGGAA	115
			Reverse	TCGTCGGCGCTTCTCAATTA	
*Hes1*	*human*	NM_005524.4	Forward	GGAAATGACAGTGAAGCACCTCC	130
			Reverse	GAAGCGGGTCACCTCGTTCATG	
*Collagen IV*	*human*	NM_000092.5	Forward	AGAGATTGCTCTGTTTGCCAC	143
			Reverse	CGGTCCCCTCTCATTCCTT	
*MMP-2*	*human*	NM_001127891.3	Forward	CTTCCAAGTCTGGAGCGATGT	119
			Reverse	TACCGTCAAAGGGGTATCCAT	
*DCN*	*human*	NM_001920.5	Forward	TCACAGAGCAGCACCTACCC	139
			Reverse	TTCACAACCAGGGAACCTTTTAAT	
*Notch1*	*human*	NM_017617.5	Forward	GGTGAACTGCTCTGAGGAGATC	150
			Reverse	GGATTGCAGTCGTCCACGTTGA	
*DLL4*	*human*	NM_019074.4	Forward	CTGCGAGAAGAAAGTGGACAGG	139
			Reverse	ACAGTCGCTGACGTGGAGTTCA	
*HBEGF*	*human*	NM_001945.3	Forward	CATCGTGGGGCTTCTCATGT	126
			Reverse	CCAGCCGATTCCTTGAGCA	
*MMP-9*	*human*	NM_004994.3	Forward	TGTACCGCTATGGTTACACTCG	97
			Reverse	GGCAGGGACAGTTGCTTCT	
*β-actin*	*mouse*	NM_007393.5	Forward	TATAAAACCCGGCGGCGCA	117
			Reverse	TCATCCATGGCGAACTGGTG	
*Fibronectin*	*mouse*	NM_010233.2	Forward	CCCTATCTCTGATACCGTTGTCC	145
			Reverse	TGCCGCAACTACTGTGATTCGG	
*Collagen IV*	*mouse*	NM_007735.2	Forward	GAACCTGGAAGAAAGGGAGAGG	123
			Reverse	GGAAGTGACTGCTTCTCCTGCA	
*LOX*	*mouse*	NM_010728.4	Forward	TGCACACACACAGGGATTGA	185
			Reverse	TGTAGCGAATGTCACAGCGT	
*DCN*	*mouse*	NM_007833.6	Forward	ACTCTCCAGGAACTTCGTGTCC	146
			Reverse	AGTCCCTGGAAGGCTCCGTTTT	

**Table 3:** Primary antibodies used in Western blotting.

Antibody	Concentration	Catalog Number	Company
ZO-1	1:1000	13663S	CST
HBEGF	1:1000	ab185555	Abcam
MMP-9	1:1000	ab76003	Abcam
β-tublin	1:1000	2148	CST
β-actin	1:1000	ab5316	Abcam

## Data Availability

All the data are contained within the article.

## Supplementary Materials

The following supporting information can be downloaded at https://www.mdpi.com/article/10.3390/antiox12111979/s1, Figure S1: extraction and identification of CEC. (**A**) Flow chart for the isolation and culture of mice CECs. (**B**) Immunofluorescence staining of CECs with CD31 and VE-cadherin Scale bars: 50 µm; Figure S2: endothelial Notch signaling activation alleviates mice CNV injuries. (**A**) In laser induced CNV model, whole-mount choroidal for EC-specific NICD overexpressing mouse CDH5-CRE-NICD (NICD) and control mice (CTRL) were analyzed using IB-4 staining. IB-4 positive areas were quantitatively compared. Scale bars: 150 µm. (**B**) mRNA levels of ECM related genes DCN, collagen IV, fibronectin and LOX in choroidal tissue were tested in EC-specific NICD overexpressing mouse CDH5-CRE-NICD (NICD) and control mice (CTRL) at CNV day7 by qRT-PCR. Three biological replicates were performed and dates are present as the mean ± SEM. NS, no significance, * *p* < 0.05, ** *p* < 0.01.
