# Supplementary material for: Endothelial Notch Signaling Regulates the Function of the Retinal Pigment Epithelial Barrier via EC Angiocrine Signaling
Source: Antioxidants (Basel). 2023 Nov 7;12(11):1979. doi: 10.3390/antiox12111979 (PMC10669439; doi:10.3390/antiox12111979)
Supplement: Supplementary file 1 [file antioxidants-2617848-supplementary.pdf › antioxidants-2617848-supplementary.pdf]

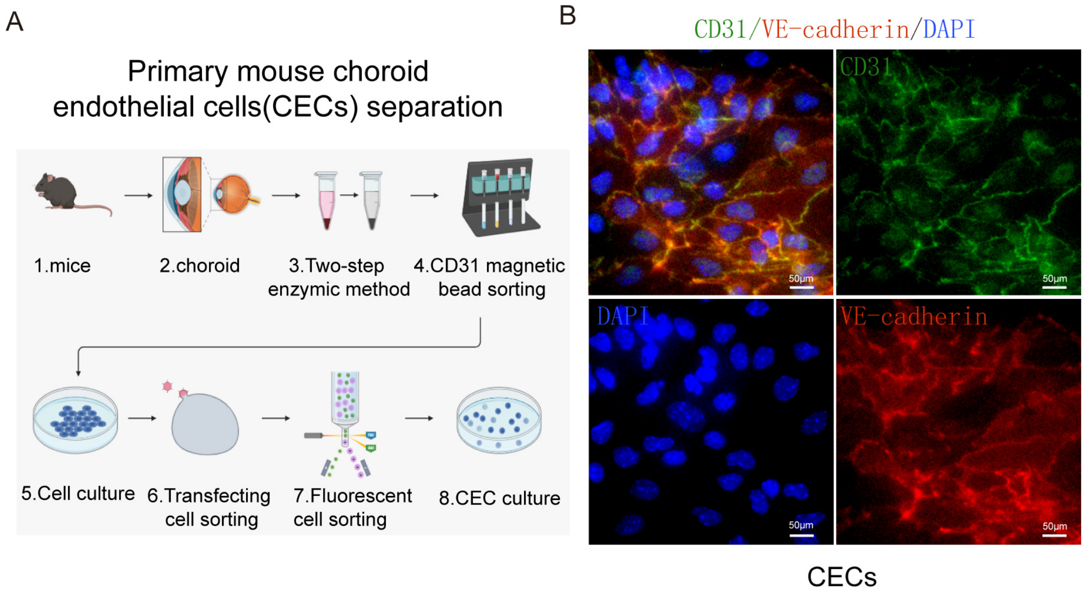

Figure S1. extraction and identification of CEC. (**A**) Flow chart for the isolation and culture of mice CECs. (**B**) Immunofluorescence staining of CECs with CD31 and VE-cadherin Scale bars: 50 μm.

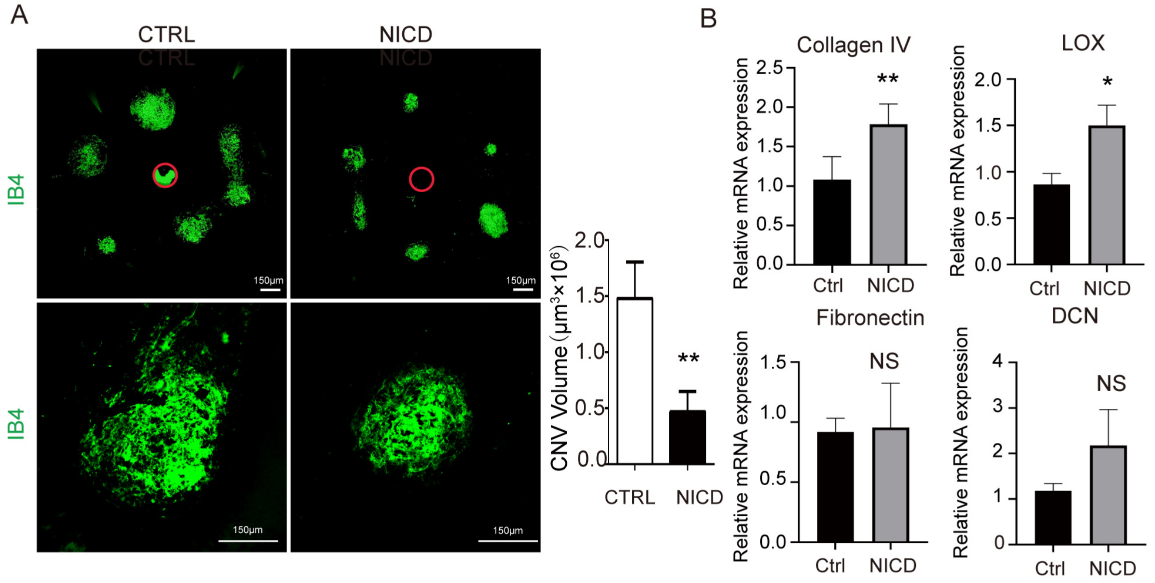

Figure S2. endothelial Notch signaling activation alleviates mice CNV injuries. (**A**) In laser induced CNV model, whole-mount choroidal for EC-specific NICD overexpressing mouse CDH5-CRE-NICD (NICD) and control mice (CTRL) were analyzed using IB-4 staining. IB-4 positive areas were quantitatively compared. Scale bars: 150 μm. (**B**) mRNA levels of ECM related genes DCN, collagen IV, fibronectin and LOX in choroidal tissue were tested in EC-specific NICD overexpressing mouse CDH5-CRE-NICD (NICD) and control mice (CTRL) at CNV day7 by qRT-PCR. Three biological replicates were performed and dates are present as the mean ± SEM. NS, no significance, * $p < 0.05$, ** $p < 0.01$.
